# STK11 Causative Variants and Copy Number Variations Identified in Thai Patients With Peutz-Jeghers Syndrome

**DOI:** 10.7759/cureus.34495

**Published:** 2023-02-01

**Authors:** Wannasiri Chiraphapphaiboon, Wanna Thongnoppakhun, Thawornchai Limjindaporn, Sunisa Sawasdichai, Ekkapong Roothumnong, Kanjana Prangphan, Benjaporn Pamornpol, Chanin Limwongse, Manop Pithukpakorn

**Affiliations:** 1 Department of Anatomy, Faculty of Medicine Siriraj Hospital, Mahidol University, Bangkok, THA; 2 Siriraj Genomics, Office of the Dean, Faculty of Medicine Siriraj Hospital, Mahidol University, Bangkok, THA; 3 Division of Medical Genetics, Department of Medicine, Faculty of Medicine Siriraj Hospital, Mahidol University, Bangkok, THA

**Keywords:** copy number variations, deletions, mutations, peutz-jeghers syndrome, stk11

## Abstract

Introduction

Peutz-Jeghers syndrome (PJS) is a rare autosomal dominant inherited disorder caused by germline mutations in the serine-threonine kinase 11 (*STK11*) tumor suppressor gene. This syndrome is characterized by hamartomatous gastrointestinal polyps, mucocutaneous melanin pigmentation, and a higher risk of developing various cancers.

Methods

We summarized the clinical and molecular characteristics of five unrelated Thai patients with PJS. Denaturing high-performance liquid chromatography (DHPLC) screening, coupled with direct DNA sequencing and multiplex ligation-dependent probe amplification (MLPA), were applied for the molecular analysis of *STK11*.

Results

A total of four *STK11 *pathogenic changeswere identified in the five PJS patients, including two frameshift variants (a novel c.199dup, p.Leu67ProfsTer96 and a known c.834_835del, p.Cys278TrpfsTer6) and two types of copy number variations (CNV), exon 1 deletion and exons 2-3 deletion. Among reported *STK11 *exonic deletions, exon 1 and exons 2-3 deletions were found to be the two most commonly deleted exons.

Conclusion

All identified *STK11 *mutations were null mutations that were associated with more severe PJS phenotypes and cancers. This study broadens the phenotypic and mutational spectrum of *STK11 *in PJS.

## Introduction

Peutz-Jeghers syndrome (PJS) is a rare autosomal dominant polyposis syndrome characterized by hamartomatous gastrointestinal polyps associated with mucocutaneous melanin pigmentations [1,2]. The estimated incidence of PJS is reported to be between 1 in 8,300 and 1 in 280,000 live births [3,4]. Patients with PJS have a 15-fold relative risk of developing various gastrointestinal and extra-gastrointestinal cancers compared to the general population, which leads to increased mortality [5]. Colorectal and breast cancers are the most common cancers reported in PJS [6,7]. In addition, complications resulting from the polyps, including intussusception, intestinal obstruction, and bleeding, are also common causes of death [3,8]. Germline mutations in the serine-threonine kinase 11 (*STK11*) tumor suppressor gene, detected in approximately 30-80% of PJS cases, have been demonstrated as the major pathogenesis [9-11]. This gene, located on chromosomal region 19p13.3, contains nine coding exons, which are translated into a 433-amino-acid protein [9,10]. *STK11* protein functions as a master upstream kinase of AMP-activated protein kinase (AMPK) signaling, which is responsible for the regulation of cell polarity, cell proliferation, and cell metabolism [12-15]. Defective STK11/AMPK activity contributes to phenotypic manifestations of PJS, including polyp formation and cancer susceptibility metabolism [12-15].

More than 500 mutations of *STK11* have been identified, and most of them result in abnormal protein truncation and loss of kinase activity [16-18]. The majority of pathogenic mutations in *STK11* are point mutations, which consist of missense/nonsense mutations (42.7%), small insertions or deletions (31.1%), and splicing mutations (7.7%) [17]. The copy number variations (CNV) associated with large genomic deletions, including exonic and whole gene deletions, account for 17% of *STK11* mutations, whereas only 1.4% of the mutations are attributable to gross insertions/ duplications, and complex rearrangements [17].

Here, we report the clinical and molecular findings of five unrelated Thai patients with PJS. Four *STK11* pathogenic variants and a CNV were identified using denaturing high-performance liquid chromatography (DHPLC), direct DNA sequencing, and multiplex ligation-dependent probe amplification (MLPA). The genotype-phenotype correlations between *STK11* mutations and PJS were also explored.

## Materials and methods

Patients and DNA extraction

The study protocol was approved by the Siriraj Institutional Review Board (COA no. Si711/2015). Five unrelated Thai patients with PJS (two males and three females) from the Department of Medicine, Faculty of Medicine Siriraj Hospital, Mahidol University, Thailand, were included in this study. All patients were diagnosed based on family history and clinical symptoms consisting of histopathologically confirmed hamartomatous polyps of PJS and mucocutaneous melanin pigmentation. Genomic DNA was extracted from peripheral blood samples by the standard salting-out method.

DNA analysis for point mutations in *STK11*

All nine coding exons of *STK11* (NM_000455.5) were amplified by polymerase chain reaction (PCR) using the primers as designed with Oligo6® software (DBA Oligo, Inc., Colorado Springs, CO) (Table 1). The PCR reactions were performed in a total volume of 25 µL containing 125 ng of genomic DNA, 5 µL of detergent-free 5× Phusion™ HF buffer containing 7.5 mM MgCl_2_, which provides 1.5 mM MgCl_2_ in the final concentration (only in exon 3, 5× Phusion™ GC buffer was used instead), 200 µM of deoxynucleotide triphosphate (dNTPs), 5 pmol of each primer, and 0.3 units of Phusion™ DNA polymerase (Finnzymes, Espoo, Finland). The PCR amplifications consisted of initial denaturation at 98 °C for 30 seconds, followed by 37 cycles of denaturation at 98 °C for 10 seconds, annealing at the optimized temperature of each exon for 20 seconds and extension at 72 °C for 30 seconds, and final extension at 72 °C for seven minutes. The PCR products (5 µL) were loaded onto 1.5% agarose gels to verify the amplicon size by comparing with the GeneRuler™ 100 bp Plus DNA ladder (Fermentas Life Science, Ottawa, ON, Canada).

**Table 1:** Primer sequences of STK11 in PCR amplification

Exon	Primer sequences (5’ to 3’)	Annealing temperature (ºC)	Amplicon size (bp)
1	F: TGGAGAAGGGAAGTCGGAAC, R: CAGCTCAGGGTGTTAAGAGGAA	62	492
2	F: TCCCACAGCACTGTGAACTC, R: CATTGCCACAATGGCTGACT	60	395
3	F: GCCTTTTCAGAGGGGTGGCT, R: GTGTGGCCTCACGGAAAGGA	72	327
4-5	F: TGTGTGCCTGGACTTCTGTGA, R: CACAGGCCGGCAGCTGCCCAA	72	555
6	F: GCAGCTCCAGGCCCCTTC, R: TCTCAATGCCTGCTGGGGTC	64	466
7	F: AGGAGTGGAGTGGCCTCTGTC, R: CCTGCTCTAGCGCCCGCTCA	62	308
8	F: AGGTACCCTGGGCCCAGA, R: TGTTGCAGACAGGCAGGCAC	62	361
9	F: CCTGGGCAGCAGCTGTAAGTG, R: CGTGACGGTCACCATGACTGA	64	420

A partially denaturing mode of DHPLC (Transgenomic, Inc., Omaha, NE, USA) was used to screen for point mutations in *STK11* by detection of heteroduplex formation. The melting temperature of each *STK11* fragment was prepared for optimized partially denaturing conditions depending on their sequence, while the suitable concentration gradient of buffer B was automatically generated by Navigator™ Software (Transgenomic, Inc.) based upon the fragment length. The amplified PCR products were denatured at 95 °C for 5 min and reannealed at a gradually decreasing temperature, resulting in heteroduplex formation. Then, 5 µL of PCR products were directly injected into the DHPLC column, and the samples were eluted with a linear gradient of buffer A (0.1 M TEAA) and buffer B (25% ACN in 0.1 M TEAA). The output data were analyzed with Navigator™ Software and displayed in chromatograms.

The fragments showing abnormal peaks were reamplified and subsequently analyzed by direct DNA sequencing. PCR products were purified using ExoSAP-IT® (Affymetrix, Santa Clara, CA, USA) and sequenced using the BigDye® Terminator v3.1 Cycle Sequencing Kit (Thermo Fisher Scientific, Waltham, MA, USA) and the Applied Biosystems® 3130xl Genetic Analyzer (Thermo Fisher Scientific). DNA sequencing was conducted in both forward and reverse orientations. All sequencing reactions were performed and analyzed alongside samples with wild-type genotypes using Applied Biosystems® Sequencing Analysis Software v.5.3.1 (Thermo Fisher Scientific).

DNA analysis for CNV in *STK11*

Semi-quantitative multiplex PCR/DHPLC was performed to determine *STK11* copy number alterations, including deletions and duplications, in the samples showing negative DHPLC-screening results. Two sets of multiplex PCRs were established. The first set included exons 2-6 of *STK11*. The second set contained exons 7-9 and exon 1 of *STK11*. Insulin-like growth factor 1 (IGF1) gene was used as an internal control for autosomal chromosomes, while acyl-CoA thioesterase 9 (ACOT9) or dystrophin (DMD) (primer sequences available on request) represented an internal control for X-chromosomes. The multiplex PCR reactions were performed in a total volume of 25 µL containing 100 ng of genomic DNA, 1× Immo buffer, 2 mM of MgCl2, 400 µM of dNTPs, 1× Q solution, 0.75 units of Immolase™ DNA polymerase (Bioline USA, Inc., Kenilworth, NJ, USA) and optimal concentrations of each primer set (Table 2). The multiplex PCR conditions consisted of initial denaturation at 95 °C for 10 seconds, followed by 25 cycles of denaturation at 95 °C for 30 seconds, annealing and extension at 60 °C for 1 minute, 30 seconds, and final extension at 60 °C for 7 seconds. The multiplex PCR products (5 µL) were loaded onto 2.5% agarose gels to verify the amplicon size by comparing with the GeneRuler™ 100 bp Plus DNA ladder (Fermentas Life Science).

**Table 2:** Two sets of multiplex PCR

Set	Gene	Amplicon size (bp)	Final concentration of primer (µM)
1	ACOT9	177	2.8
	IGF1	267	3.5
	STK11 Exon 3	327	4.5
	STK11 Exon 2	395	8.0
	STK11 Exon 6	466	6.0
	STK11 Exon 4-5	555	7.0
2	DMD	171	4.2
	IGF1	267	2.5
	STK11 Exon 7	308	6.0
	STK11 Exon 8	361	4.0
	STK11 Exon 9	420	8.0
	STK11 Exon 1	492	15.0

Non-denaturing mode of DHPLC was applied for the separation of multiple DNA fragments from multiplex PCR reactions depending on their sizes. The aliquots (10 µL) of multiplex PCR products were injected into a DHPLC system, which maintained the column temperature at 50 ºC. A normal control sample of the same gender was always injected in the same run to compare the results. The DHPLC peak heights reflect the quantity of PCR products in each DNA fragment.

The samples showing abnormal peak heights were also verified for copy number alterations using MLPA. The MLPA reactions were performed using SALSA® MLPA® probemix P101-B3 *STK11* (MRC-Holland, Amsterdam, The Netherlands) following the manufacturer’s recommendations and analyzed using an Applied Biosystems® 3130xl Genetic Analyzer (Thermo Fisher Scientific) and Coffalyser v.140721.1958 software (MRC-Holland).

## Results

A mutation analysis was performed in five unrelated PJS patients. All patients presented with mucocutaneous pigmentation, gastrointestinal polyposis, and polyp-related complications (Table 3). Three of five patients had a documented family history of PJS. None of the gastrointestinal cancers have been observed, but two gynecological cancers, including sex cord tumorlets with annular tubules (SCTAT), cervical mucinous adenocarcinoma, and papillary breast cancer, were reported in three female patients.

**Table 3:** STK11 mutations and clinical characteristics of PJS patients PJS: Peutz-Jeghers syndrome, F: female, M: male, Lp: lips, Bm: buccal mucosa, Hd: hands, Ft: feet, St: stomach, Sb: small bowel, Lb: large bowel, SCTAT: sex cord tumorlets with annular tubules

Patient	Sex	Age of diagnosis	Family history	Pigmentation (location)	Polyp (location)	Non-malignant symptom (× number)	Cancer	Mutation
PJS01	F	22	No	Yes (Lp-Hd)	Yes (St-Sb-Lb)	Abdominal pain, bloody stools	No	Del. exon 1
PJS02	M	13	Yes	Yes (Lp-Bm-Hd)	Yes (St-Sb-Lb)	Abdominal pain, bloody stools, anemia, intussusception (×3), small bound obstruction	No	Del. exon 1
PJS03	F	18	Yes	Yes (Lp-Bm)	Yes (St-Sb-Lb)	Abdominal pain, bloody stools, anemia, intussusception (×2)	SCTAT	c.199dup (p.L67Pfs*96)
PJS04	F	32	No	Yes (Lp-Bm-Hd-Ft)	Yes (St-Sb-Lb)	Abdominal pain, bloody stools, anemia, intussusception (×1)	Cervical mucinous adenocarcinoma	Del. exons 2-3
PJS05	F	5	Yes	Yes (Lp-Bm)	Yes (St-Sb-Lb)	Abdominal pain, bloody stools, anemia, intussusception (×2)	Papillary breast cancer	c.834_835del (p.C278Wfs*6)

**Figure 1:**
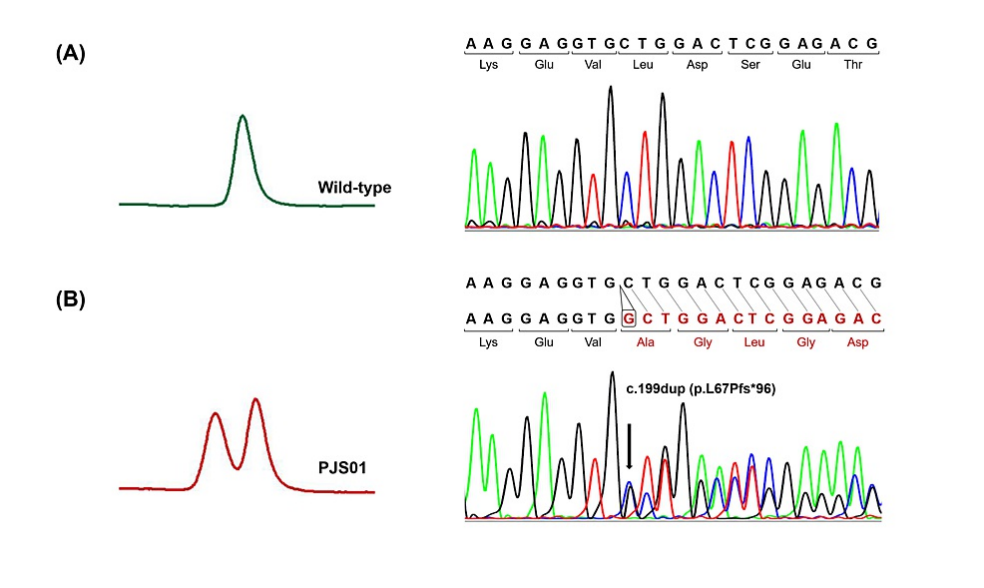
DNA analysis of PJS03 showing c.199dup (p.Leu67ProfsTer96) mutation in STK11 (A) wild-type control. (B) PJS03 patient. A heteroduplex formation of exon 1 fragment in DHPLC analysis (left panel) relates to a heterozygous 1-bp duplication of guanine (G) locating after the nucleotide 199 in sequencing analysis (right panel). The mutation results in a translational frameshift starting from codon 67, generating a premature stop codon at codon 162 in exon 4. The identified mutation is marked by an arrow.

**Figure 2:**
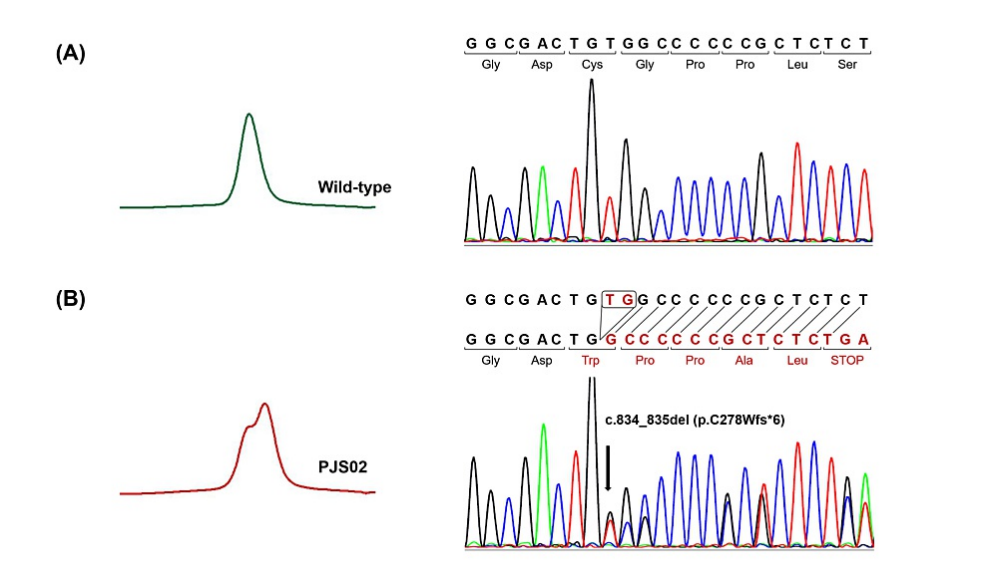
DNA analysis of PJS05 showing c.834_835del (p.Cys278TrpfsTer6) mutation in STK11 (A) Wild-type control and (B) PJS05 patient. A heteroduplex formation of exon 6 fragment in DHPLC analysis (left panel) relates to a 2-bp deletion locating between nucleotides 834 and 835 in sequencing analysis (right panel). The mutation results in a translational frameshift starting from codon 278, generating a premature stop codon at codon 283 in exon 6. The identified mutation is marked by an arrow.

Four pathogenic *STK11* variants/CNV were identified in all five patients, including one deletion, one duplication, and two exonic deletions (Table 4). Abnormal heteroduplex peaks were detected in two patients, PJS03 and PJS05, under a partially denaturing condition of DHPLC. Accordingly, a novel heterozygous 1-bp duplication (c.199dup, p.Leu67ProfsTer96) and a reported heterozygous 2-bp deletion (c.834_835del, p.Cys278TrpfsTer6) were confirmed in PJS03 and PJS05 by direct DNA sequencing, respectively (Figures 1-2).

**Table 4:** Interpretation of STK11 variants and copy number variations identified in this study

No.	STK11 variants/CNV	ClniVar ID	Classification	Criteria met	References
1	NM_000455.5:c.199dup, p.Leu67ProfsTer96	None	Pathogenic	PVS1, PM2, PP4	[19]
2	NM_000455.5:c.834_835del, p.Cys278TrpfsTer6	7444	Pathogenic	PVS1, PM2, PP4, PP5	[19]
3	Exon 1 deletion: NC_000019.9:g.(?_1206913)_(1207212_?)del	1073837	Pathogenic	2C-1 (1.00) + 5H (0.30) = 1.30	[20]
4	Exon 2-3 deletion: NC_000019.9:g.(?_1218406)_(1219422_?)del	1073840	Pathogenic	2E (0.90) + 5H (0.30) = 1.20	[20]

The pathogenicity interpretation of the identified *STK11* variants and CNV was performed (as shown in Table 4) according to the 2015-standards and guidelines recommended by the American College of Medical Genetics and Genomics (ACMG) and the Association for Molecular Pathology (AMP) [19], as well as the 2019 technical standards for the CNV interpretation recommended by the ACMG and the Clinical Genome Resource (ClinGen) [20], respectively.

**Figure 3:**
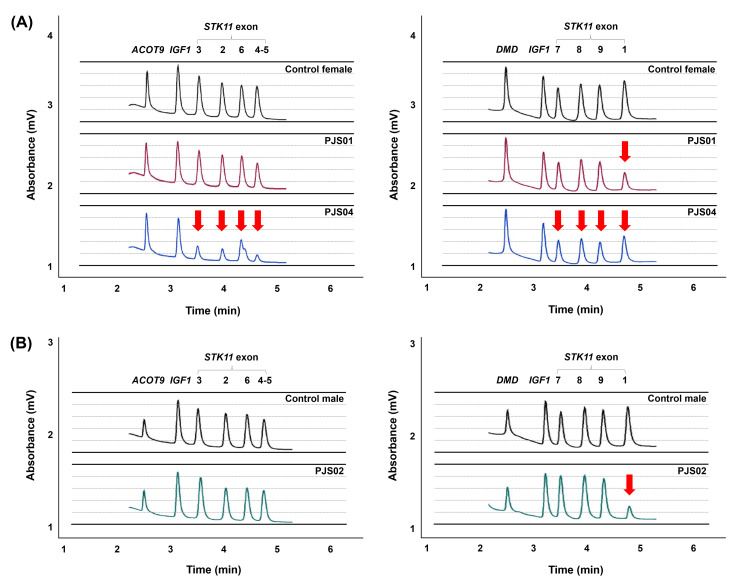
Denaturing high-performance liquid chromatography profiles of two multiplex PCR sets showing copy number alterations of STK11 (A) PJS01 (red chromatogram) and PJS04 (blue chromatogram) patients and (B) PJS02 patient (green chromatogram). The left and right panels represent multiplex PCR set 1 and 2, respectively. Genes and exons under study are indicated on the top. The chromatograms in black represent normal control samples of the same gender with patients. The identified exonic deletions are marked by an arrow.

**Figure 4:**
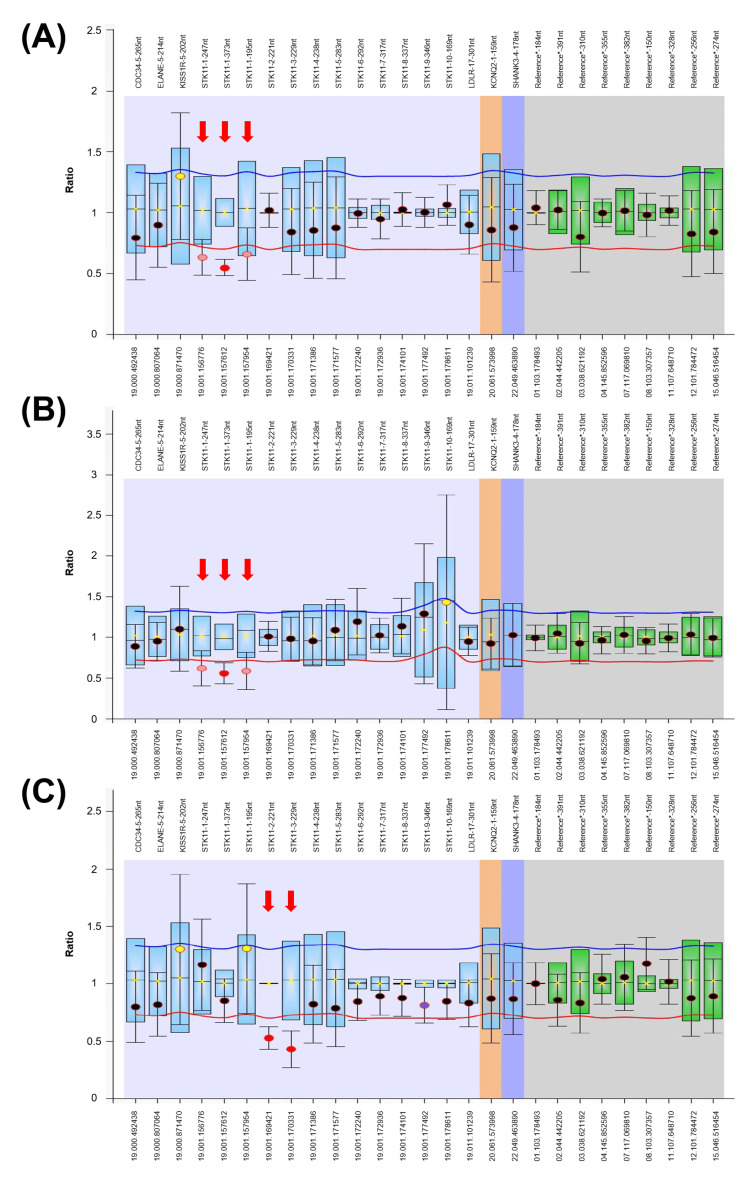
Multiplex ligation-dependent probe amplification analysis showing exonic deletions of STK11 (A) PJS01 patient (exon 1 deletion) and (B) PJS02 patient (exon 1 deletion). (C) PJS04 patient (exons 2-3 deletion). The X-axis shows the chromosomal locations and the Y-axis shows dosage quotient (DQ). The probe names are indicated on the top. The normal exon dosage is ranging from 0.7 to 1.3. The identified exonic deletions are marked by an arrow.

The samples showing no heteroduplex formation in DHPLC screening results were subsequently determined for *STK11* copy number alterations using semi-quantitative multiplex PCR/DHPLC. The exon 1 deletion was identified in PJS01 and PJS02. Meanwhile, DHPLC peaks dropped in all exons of *STK11* were found in PJS04 (Figure 3). MLPA analysis was used to confirm previously detected exon deletions. The exon 1 deletion was found in PJS01 and PJS02 and agrees with DHPLC analysis, but the exons 2-3 deletion was identified in PJS04, and the result is inconsistent with the whole gene deletion in DHPLC analysis (Figure 4). Although the DHPLC results demonstrated the whole deletion of STK11 in PJS04, they were incompatible when analyzed with the sequencing results. Two heterozygous single base changes were identified in intron 7 (c.920+7G>C) and intron 9 (c.*16+45T>A) of *STK11* in PJS04 (data not shown). Moreover, the incorporation of another unrelated internal control gene, proline-rich transmembrane protein 2 (PRRT2), with a large amplicon size of 640 bps in multiplex PCR revealed the reduction of DHPLC peaks, which indicated the effect of DNA degradation in PJS04 (data not shown). Using all mutation analysis together, the mutation of PJS04 was therefore concluded to be exons 2-3 deletion of *STK11*.

## Discussion

PJS is a rare inherited disorder in which *STK11* is known as the causative gene [9-11]. Although mutation studies of PJS have been conducted in many countries, there are very few studies of PJS in Thailand, and the genetics of this syndrome remain obscure. This study attempts to investigate the clinical and molecular characteristics of Thai PJS patients and apply the versatile DHPLC method to perform the mutation analysis of *STK11*.

Using DHPLC-based heteroduplex screening and direct DNA sequencing, two *STK11* point mutations were identified. A 1-bp frameshift duplication (c.199dup, p.Leu67ProfsTer96) was found in PJS03 (Figure 1). This mutation has never been reported in PJS; however, the early termination caused by this mutation (p.L67Pfs*96) was similar to the effect of a previously reported adjacent mutation (c.198dup), which clearly cosegregated with PJS in the original family of Peutz’s study [21]. Both the PJS03 patient and affected members of the original PJS family presented with gastrointestinal polyps, mucocutaneous pigmentation, and cancer development. Nonetheless, there were different manifestations of polyp location and cancer types. SCTAT, a rare ovarian cancer associated with PJS [22], was identified in PJS03. On the other hand, gastrointestinal cancers, colon and gastric cancers, and breast cancer were found in the original PJS family [21]. There were unusual nasal polyps that developed in 6 out of 22 PJS patients of the original PJS family while being absent in PJS04 [21]. Another point mutation is a 2-bp frameshift deletion (c.834_835del, p.C278Wfs*6) found in PJS05 (Figure 2). This mutation has previously been noted in one case of PJS [9], but there was no reported clinical manifestation related to the mutation.

Semi-quantitative multiplex PCR/DHPLC and MLPA analysis revealed two exonic deletions of *STK11*. The exon 1 deletion was identified in PJS01 and PJS02. While the exons 2-3 deletion was confirmed in PJS04, the different results between DHPLC and MLPA were observed. All DHPLC peaks of the *STK11* amplicons decreased, but MLPA indicated exons 2-3 deletion. Two heterozygous variants found in intron 7 (c.920+7G>C) and intron 9 (c.*16+45T>A) of *STK11* in PJS04 indicated that the whole gene deletion might not be possible and the DHPLC peak reduction might be caused by other reasons. It was noticed that decreased DHPLC peaks were also found in large-sized PCR amplicons of unrelated PRRT2 genes in PJS04, suggesting the effect of poor DNA quality. Degraded DNA might disrupt semi-quantitative multiplex PCR/DHPLC analysis to complete the DNA profile or produce an inaccurate result. On the other hand, MLPA probes are able to hybridize small target sequences (50-70 nucleotides), providing better tolerance for the analysis of fragmented DNA. These ambiguous results suggested that MLPA, the present standard method to evaluate copy number alterations in *STK11*, remains needed to prove the correctness of semi-quantitative multiplex PCR/DHPLC results.

Large genomic deletions are common *STK11* mutations in PJS patients apart from point mutations. According to the published reports, the deletions often involve the whole gene or are distributed in the 5' upstream region of *STK11*. Noticeably, exon 1 and exons 2-3 represent the most frequently deleted exons that correlate to the exonic deletions found in our patients (Table 5). An overrepresentation of the Alu repetitive sequence has been proposed as a potential mechanism that mediates homologous recombination, leading to recurrent genomic rearrangements and copy number alterations in *STK11* [23,24]. Specifically, Alu elements were highly detected in intron 1 and breakpoint characterization of PJS patients harboring *STK11* exons 2-3 deletion indicated that Alu elements located within intron 1 and 3 serve as recombination hotspots [23,24]. Other Alu elements were also predicted to mediate exon 2 and exons 1-3 deletions [24]. These data suggested that exon 1 deletion might occur via the same mechanism.

**Table 5:** The distribution of reported copy number variations in STK11 found as exonic and whole gene deletions X represents promotor/exon deletion. P represents partial exon deletion^f^. ^a^Non-coding exon; ^b^whole gene deletions; ​​​​​​​^c^two cases have chromosome 19p13.3 deletion; ​​​​​​​^d^some cases have deletions of the adjacent genes including *SBNO2*, *GPX4*, *POLR2E*, *ABCA7*, *C19orf26*, *ATP5D*, *MIDN*, *C19orf23*, *CIRBP*, *C19orf24* and *EFNA2; *^e^alu-mediated homologous recombination is presumed to be one mechanism of deletions; ​​​​​​​^f^partial exon deletions include the deletions occurred in exonic regions that have the deleted bases more than 20 bases.

Promoter	STK11 exon(s)										Case number	References
	1	2	3	4	5	6	7	8	9	10^a^		
	X										30	[18,26,27,30-36]
X	X	X	X	X	X	X	X	X	X	X	21^b,c,d^	[30,31,33,34,37-43]
		X	X								17^e^	[9,23,24,27,30-34,40,44-46]
X	X										8	[38,40,44]
		X	X	X	X	X	X	X	X	X	6	[30,31,38,44]
								X			5	[9,27,30,38,47]
	X	X	X								3^e^	[24,31-33]
							X				3	[46]
X	X	X	X	X	X	X	X				2^c^	[32,33]
		X									2^e^	[24,34,39]
		X	X	X	X						2	[36]
				X	X	X	X	X			2	[48]
			X								1	[18]
						X					1	[18]
X											1	[38]
X	X	X	X								1	[38]
X	X	X	X	X	X						1^c^	[33]
			X	X	X	X	X				1	[32]
			X	X	X	X	X	X	X		1	[18]
		X	X	X	X	X	X				1	[39]
		X	X	X	X	X	X	P			1	[49]
		X	X	X	X	X	X	X			1	[50]
		X					X	X	X	X	1	[40]
				X	X						1	[30]
				X	X	X					1	[18]
						X	X	X			1	[31]
							X	X			1	[31]
								X	X		1	[44]
				P							3	[6,51,52]
			P								3	[6,51,53]
	P										2	[9,25]
							P				1	[54]

The genotype-phenotype correlation between *STK11* mutations and PJS has been investigated in several studies. The *STK11* frameshift mutations in PJS03 and PJS05 led to premature termination, resulting in a truncated protein with a disrupted catalytic kinase domain and a complete absence of the C-terminal regulatory region. The *STK11* exonic deletions, including exon 1 in PJS01 and PJS02 and exons 2-3 in PJS04, also affected the kinase domain, resulting in the inactivation of *STK11* kinase activity. Null STK11 mutations, including both truncating mutations and large deletions, have found to be associated with more severe phenotypes of PJS. Patients with null *STK11* mutations tend to have early-onset, frequent gastrointestinal symptoms including polyps, intussusception, and intestinal obstruction compared to patients with missense mutations [11,25-27]. While previous studies showed the median age of diagnosis and first gastrointestinal symptom onset in patients with null *STK11* mutations to be 17 and 10 years, respectively [25,26], the median diagnostic age of our patients was 18 years, with intussusception as the most frequent first symptom. In terms of cancer development, there was no difference in cancer risks between null mutations and missense mutations; however, breast cancer was associated with truncating mutations [7,26,28]. Three of the five patients in our study developed cancer, and one patient who carried a truncating mutation, PJS05, also presented with papillary breast cancer. Two rare PJS-related gynecological cancers were diagnosed in the other two patients, including SCTAT in PJS03 and cervical mucinous adenocarcinoma in PJS04. No obvious correlation between these two rare cancers and germline *STK11* mutations in PJS has been described; nevertheless, somatic *STK11* mutations were detected in approximately 20% of cervical cancers, and half of the harbored mutations were large monoallelic/biallelic deletions [29].

## Conclusions

In summary, we report the clinical characteristics and pathogenic *STK11* variants, or CNV, of five Thai patients with PJS. Four null mutations, including two *STK11* frameshift variants (a novel c.199dup, p.Leu67ProfsTer96, and a known c.834_835del, p.Cys278TrpfsTer6), and two common STK11 exonic deletions, exon 1 deletion and exons 2-3 deletion, were identified. These *STK11* mutations were associated with more severe manifestations of PJS. The molecular analysis of the *STK11* gene using the rapid and cost-effective DHPLC method for mutation screening combined with standard direct DNA sequencing and MLPA is well optimized and demonstrated. This study expands knowledge on the molecular genetics of *STK11* mutations, which is beneficial for clinical diagnosis and genetic counseling.
